# Supplementary material for: Anti-Inflammatory Activity of Four Triterpenoids Isolated from Poriae Cutis
Source: Foods. 2021 Dec 20;10(12):3155. doi: 10.3390/foods10123155 (PMC8700795; doi:10.3390/foods10123155)
Supplement: Supplementary file 1 [file foods-10-03155-s001.zip › foods-1466945-supplementary.pdf]

Supplementary informations

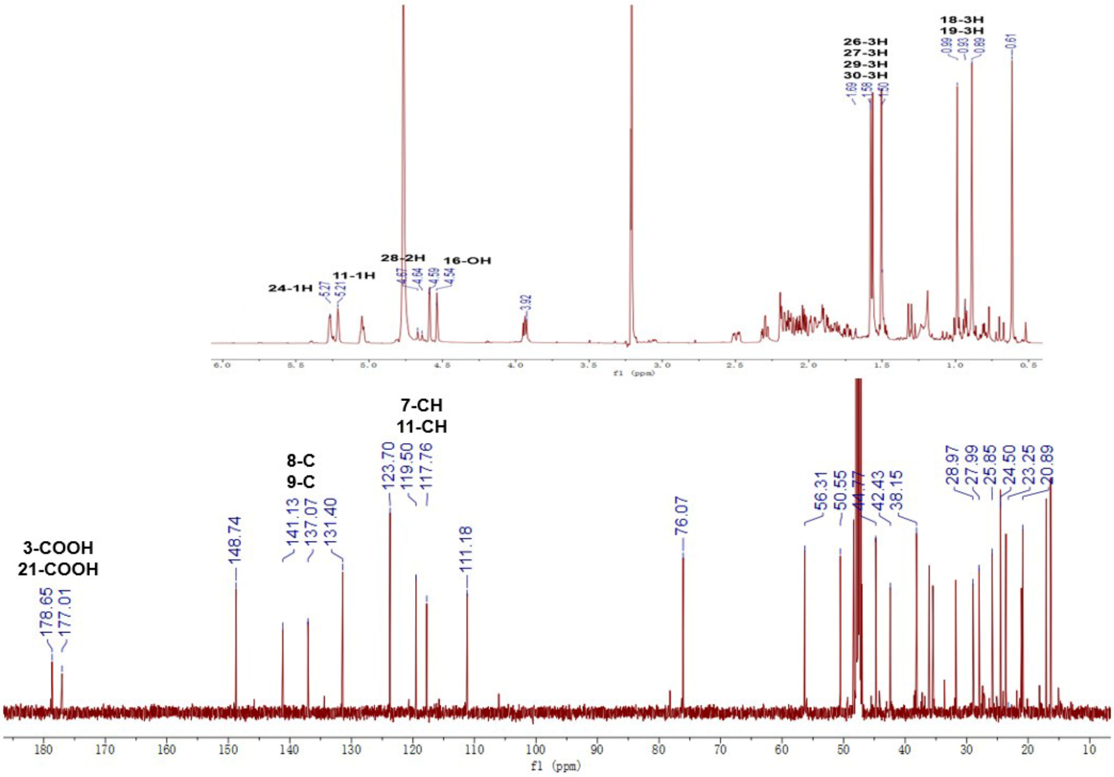

Figure S1. ^{1}H- and ^{13}C-NMR spectra of compound 1.

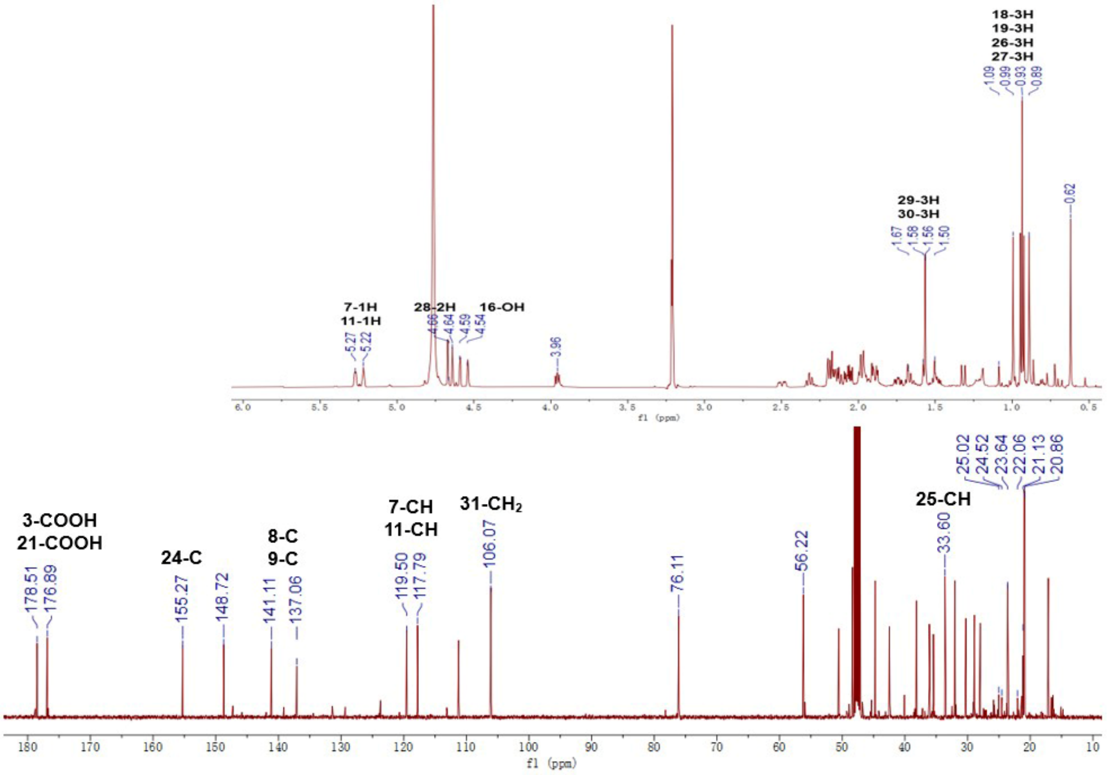

Figure S2. ^{1}H- and ^{13}C-NMR spectra of compound 2.

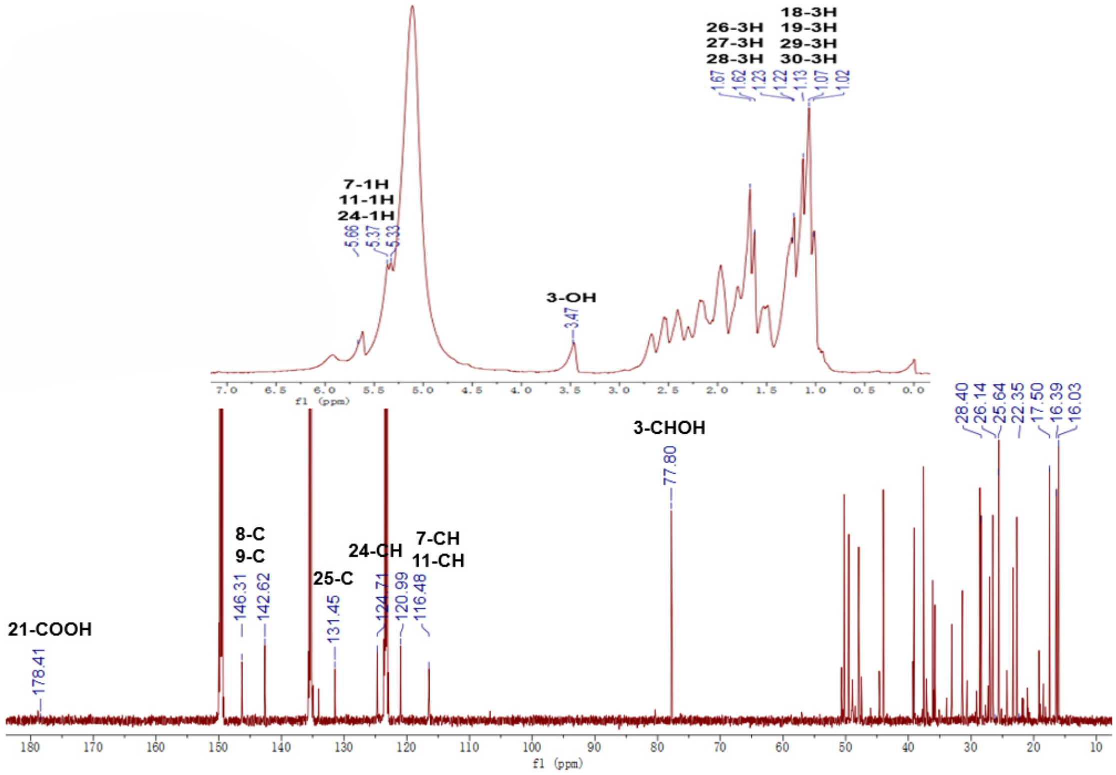

Figure S3. ^{1}H- and ^{13}C-NMR spectra of compound 3.

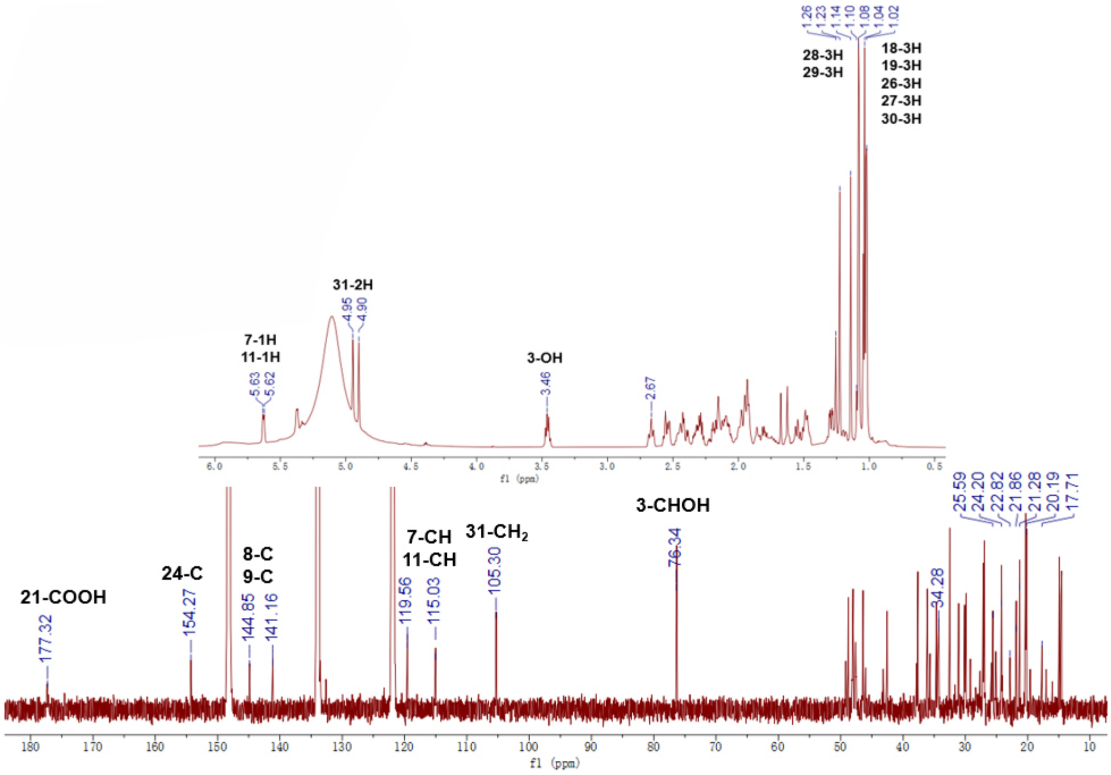

Figure S4. ^{1}H- and ^{13}C-NMR spectra of compound 4.
